# Variants at the *ASIP* locus contribute to coat color darkening in Nellore cattle

**DOI:** 10.1186/s12711-021-00633-2

**Published:** 2021-04-28

**Authors:** Beatriz B. Trigo, Adam T. H. Utsunomiya, Alvaro A. A. D. Fortunato, Marco Milanesi, Rafaela B. P. Torrecilha, Harrison Lamb, Loan Nguyen, Elizabeth M. Ross, Ben Hayes, Rômulo C. M. Padula, Thayla S. Sussai, Ludmilla B. Zavarez, Rafael S. Cipriano, Maria M. T. Caminhas, Flavia L. Lopes, Cassiano Pelle, Tosso Leeb, Danika Bannasch, Derek Bickhart, Timothy P. L. Smith, Tad S. Sonstegard, José F. Garcia, Yuri T. Utsunomiya

**Affiliations:** 1 School of Veterinary Medicine, Araçatuba, Department of Production and Animal Health, São Paulo State University (Unesp), Araçatuba, SP Brazil; 2 International Atomic Energy Agency (IAEA) Collaborating Centre On Animal Genomics and Bioinformatics, Araçatuba, SP Brazil; 3 AgroPartners Consulting, R. Floriano Peixoto, 120-Sala 43a-Centro, Araçatuba, SP 16010-220 Brazil; 4 Personal-PEC, R. Sebastião Lima, 1336-Centro, Campo Grande, MS 79004-600 Brazil; 5 Centre for Animal Science, Queensland Alliance for Agriculture and Food Innovation, The University of Queensland, Brisbane, Australia; 6 Centro Universitário Católico Salesiano, Araçatuba, SP Brazil; 7 CRV Lagoa, Sertãozinho, SP Brazil; 8 Institute of Genetics, Vetsuisse-Faculty, University of Bern, Bremgartenstrasse 109A, 3012 Bern, Switzerland; 9 Dermfocus, University of Bern, Bremgartenstrasse 109A, 3012 Bern, Switzerland; 10 Department of Population Health and Reproduction, School of Veterinary Medicine, University of California, Davis, CA 95616 USA; 11 Dairy Forage Research Center, USDA-ARS, 1925 Linden Drive, Madison, WI 53706 USA; 12 US. Meat Animal Research Center, USDA-ARS, 844 Road 313, Clay Center, NE 68933 USA; 13 Recombinetics Inc., St. Paul, MN USA; 14 School of Agriculture and Veterinarian Sciences, Jaboticabal, Department of Preventive Veterinary Medicine and Animal Reproduction, São Paulo State University (Unesp), Jaboticabal, SP Brazil

## Abstract

**Background:**

Nellore cattle (*Bos indicus*) are well-known for their adaptation to warm and humid environments. Hair length and coat color may impact heat tolerance. The Nellore breed has been strongly selected for white coat, but bulls generally exhibit darker hair ranging from light grey to black on the head, neck, hump, and knees. Given the potential contribution of coat color variation to the adaptation of cattle populations to tropical and sub-tropical environments, our aim was to map positional and functional candidate genetic variants associated with darkness of hair coat (DHC) in Nellore bulls.

**Results:**

We performed a genome-wide association study (GWAS) for DHC using data from 432 Nellore bulls that were genotyped for more than 777 k single nucleotide polymorphism (SNP) markers. A single major association signal was detected in the vicinity of the *agouti signaling protein* gene (*ASIP*). The analysis of whole-genome sequence (WGS) data from 21 bulls revealed functional variants that are associated with DHC, including a structural rearrangement involving *ASIP* (*ASIP*-SV1). We further characterized this structural variant using Oxford Nanopore sequencing data from 13 Australian Brahman heifers, which share ancestry with Nellore cattle; we found that this variant originates from a 1155-bp deletion followed by an insertion of a transposable element of more than 150 bp that may impact the recruitment of *ASIP* non-coding exons.

**Conclusions:**

Our results indicate that the variant *ASIP* sequence causes darker coat pigmentation on specific parts of the body, most likely through a decreased expression of *ASIP* and consequently an increased production of eumelanin.

**Supplementary Information:**

The online version contains supplementary material available at 10.1186/s12711-021-00633-2.

## Background

Brazil is the largest exporter, second largest producer, and third largest consumer of beef in the world [1]. Approximately 80% of the beef cattle in Brazil are Nellore. This *Bos indicus* cattle breed is native to the Nellore-Ongole region in the east coast of India and has been imported by many tropical countries since the late nineteenth century [2]. These imports were motivated by the adaptation of Nellore cattle to the challenging conditions of tropical and sub-tropical climates, since the breed is resistant to several diseases, survives on low-quality forage, and tolerates heat [3].

Length of the hair coat, skin pigmentation and coat color are traits that are often hypothesized to contribute to heat tolerance in mammals [4, 5]. In fact, the mixture of white/grey and dark hair that is short (5 to 8 mm average length), thick (> 50 mm), dense (> 1400 hair/cm^–2^) and placed against a black skin, provides higher reflectance at shorter light wavelengths (of particular interest in tropical regions) in Nellore than in European *Bos taurus* cattle breeds [6]. However, Nellore cattle exhibit variation in coat color patterns that is associated with sex, age, and genetic background.

Nellore cattle have black skin with cows presenting a near-white hair coat and bulls generally exhibiting darker hair ranging from light grey to black, especially on the head, neck, hump, and knees. This observation points to sex as a first source of phenotypic variation. Curiously, some bulls also have a near-white coat that resembles that of females, which is further evidence that variation in darkness of hair coat (DHC) in males is genetically determined. The parts of the body of the adult animal that present darker hair are generally reddish from birth to yearling, which suggests a melanocyte maturation process linked to puberty. Hair coat color seems to continue to darken throughout the adult life, an aging-related phenomenon that is speculated to be regulated by steroid hormones and the prolactin receptor in hair follicles [7]. However, the molecular mechanisms and genetic/epigenetic factors that underlie the observed sexual dimorphism, the variation in DHC between bulls, and the age-related darkening of hair coat in males remain uncharacterized in the Nellore breed. Given the potential complexity of coat color patterns in Nellore cattle and the putative existence of different molecular pathways governing each one of the three mentioned sources of variation, we hypothesize that they are better understood as separate, intermediate traits.

Several *B. indicus* breeds exhibit coat color patterns that are similar to those observed in Nellore cattle. Examples include the Indian-Pakistani breeds Tharparkar, Bhagnari, Dajal, Hariana, Guzerat and Ongole (from which Nellore are derived). Similar patterns are also seen in other cattle populations derived from Ongole that are reared in the Americas and Oceania, such as the Brazilian Tabapuã, the Indonesian Peranakan Ongole, and the Australian and American Brahman. In addition, several Central and Eastern European *B. taurus* breeds also display white/grey hair-coat, some of which have been reported to carry haplotypes of *B. indicus* ancestry [8, 9]. This is the case of the Chianina, Corsa, Croatian Podolian, Garfagnina, Gascon, Guelmoise, Hungarian Grey, Italian Podolian, Marchigiana, Maremmana, Piedmontese, Romagnola, Turkish Grey, Tyrolean Grey and Ukrainian Grey breeds, among others. Therefore, it is likely that common genetic variants explain this coat color pattern across breeds with shared ancestry.

Senczuk and colleagues [10] performed a genetic divergence analysis in which the above-mentioned *B. taurus* breeds were contrasted with four non-grey northwestern cattle populations (Angus, Charolais, Limousin, and Holstein), and suggested that three loci may be linked to white/grey hair-coat: CHR2:6,510,630–7,010,630, CHR14:22,531,305–25,722,332 and CHR26:22,789,524–23,289,524 based on the ARS-UCD1.2 bovine genome assembly [11]. Holland [12] also mapped white/grey coat color in a Nellore-Angus crossbred population to chromosome 6, within a segment that harbors genes known to impact coat color such as *v-kit Hardy-Zuckerman 4 feline sarcoma viral oncogene homolog* (*KIT*).

Since coat color variation may contribute to the adaptation of cattle populations to tropical and sub-tropical environments, and given the interesting complexity of the coat color phenotype in the Nellore breed, our aim was to map positional and functional candidate genetic variants associated with DHC in Nellore bulls. As argued above, the age-related darkening of hair coat in males and the sexual dimorphism component should be considered as separate traits for the sake of simplicity, and thus are the focus of separate ongoing studies.

## Methods

### Phenotypes

In total, 432 bulls were included in our genome-wide association study (GWAS). Photographs of the adult bulls, which were taken while they were on artificial insemination stations for semen sample collection, were inspected by six evaluators. Each animal was assigned a visual score based on a subjective scale ranging from 0 to 3. The lowest score (0) corresponded to completely white animals and the highest score (3) to animals with extremely dark hair on the head, neck, hump, and knees. The scores of the six evaluators were averaged to achieve a final score that was adopted as the response variable in our GWAS screening. The distribution of the resulting phenotypes is in Additional file 1: Figure S1.

### Genotypes

Illumina® BovineHD BeadChip (777 k chip) genotypes from previous studies [13, 14] were available for all the bulls. The pre-commercial single nucleotide polymorphism (SNP) panel included 786,799 markers. Prior to the association analysis, the PLINK v1.9b4.6 [15, 16] software was used to exclude SNPs with a call rate lower than 90% or a minor allele frequency lower than 2%. All bulls had a minimum genotype call rate of 90%.

### Genome-wide association study

Phenotypes were regressed onto genotypes using the mixed linear model analysis with the leave-one-chromosome-out (MLMA-LOCO) procedure in the GCTA v.1.90.2 beta software [17, 18]. This procedure accounts for population structure using the genomic relationship matrix between individuals. The inflation factor of the squared test statistics was measured as the slope of a linear regression between observed and theoretical quantiles in R version v3.6.2 [19]. The GWAS was performed a second time including the fixed effect of the top scoring SNP to test for allelic and gametic phase heterogeneity, i.e., for association signals that are driven by multiple underlying causal variants that are either independent or only partially correlated with each other.

### Test for the presence of a dominance effect

The top scoring SNP in the GWAS screening was tested for the presence of a dominance effect. Following Falconer and Mackay [20], the model below was fitted to the data using ordinary least squares regression:

$${\mathbf{y}} = {\mathbf{1}}\mu + {\mathbf{m}}\alpha + {\mathbf{w}}d + {\mathbf{e}},$$

where $\alpha$ is the allele substitution effect, $d$ is the dominance effect, and $\mathbf{m}$ and $\mathbf{w}$ are vectors relating $\mathbf{y}$ to $\alpha$ and $d$, respectively. For vector $\mathbf{m}$, genotypes 0, 1 and 2 were re-coded as $0-2p$, $1-2p$ and $2-2p$, respectively, where $p$ is the frequency of the counted allele. Conversely, vector $\mathbf{w}$ assumed values $-2{p}^{2}$, 2*pq* and $-2{q}^{2}$ for genotypes 0, 1 and 2, respectively, where $q=1-p$. In this setting, $\alpha$ represented the average effect of extra copies of the counted allele, which is a function of both additive and dominance genetic effects, i.e. $\alpha =a+(q-p)d$. This model is convenient because $COV[\mathbf{m},\mathbf{w}]=0$, thus avoiding co-linearity in the simultaneous estimation of additive and dominance effects [21]. The coefficient of determination (${R}^{2}$) of this regression model was adopted as a proxy for the proportion of phenotypic variance explained by the tested SNP. This model was fitted to the data in R version v3.6.2 [19].

### Analysis of haplotype diversity

To assess haplotype diversity at candidate loci, chromosomes that presented evidence of association with DHC were subjected to phase inference with the software Eagle v2.4.1 [22]. Then, we used the GHap v2.0.0 R package [23] to extract haplotype alleles within the chromosomal segments that presented peak associations. The distribution of phenotypes conditional on haplotypes was then inspected using boxplots in R version v3.6.2 [19].

### Whole-genome sequences of Nellore bulls

Whole-genome sequencing (WGS) data were available for 17 Nellore bulls from a previous study [14]. These animals had been sequenced on an Illumina HiSeq 2000 instrument at an average coverage of ~ 9 × using paired-end reads of 100 bp. Since none of these sequenced bulls had an average phenotype score of zero, the data was complemented with additional sequences from four white bulls that were not included in the GWAS. DNA samples from these animals were processed with the TruSeq Nano library preparation kit (Illumina) and then sequenced at ~ 10 × coverage on the Illumina Novaseq6000 platform using paired-end reads of 150 bp. All 21 bulls had their paired-end reads aligned against the ARS-UCD1.2 bovine genome assembly [11] with the Burrows-Wheeler Alignment (BWA) mem algorithm [24]. Optical and PCR duplicates were marked with the PicardTools v1.119 software (available at: http://broadinstitute.github.io/picard/). Single nucleotide variants (SNVs) and small insertions-deletions (INDEL) were extracted from aligned reads using the mpileup algorithm from SAMtools v1.9 and BCFtools v1.10.2 [25]. Variant effects were predicted and annotated with the Ensembl Variant Effect Predictor tool (VEP) [26]. Structural variants (SV) were inferred with the Gaussian Mixture Model implemented in the CNVcaller toolkit [27].

### Identification of putative causal variants

Sequence variants within each candidate region were further tested for associations with phenotypes using ordinary least squares regression in R version v3.6.2 [19]. Because of the limited sample size (21 animals), the resulting *p*-values were used only as auxiliary indicators for the location of putative causal variants. The Integrative Genomics Viewer (IGV) software [28] was used to visually confirm the existence of candidate variants and manually curate genotype calls made by CNVcaller, and to reveal additional structural variants that might have remained undetected by the described bioinformatics pipeline.

### Simulation of candidate structural variants

We performed a series of trial-and-error simulations of short-read data and of mutant genomes based on the ARS-UCD1.2 bovine assembly. For the simulation of short reads, the suspected structural arrangements were created in FASTA files by manually editing the ARS-UCD1.2 sequence. The resulting files were used to generate simulated paired-end reads with the wgsim v0.3.1-r13 program from SAMtools [25]. In the second set of simulations, the modified FASTA files were used as the reference genome, and real sequence reads were aligned against the simulated mutant genome. Different structural arrangements were tested until the pattern of read alignments in the simulations became indistinguishable from that of the empirical data. All alignments were performed with the BWA mem algorithm [24].

### Nanopore sequences of Brahman heifers

To be able to evaluate independently candidate structural variants identified in the Nellore sequence data, we produced Oxford Nanopore Technologies (ONT) sequences from DNA extracted from the tail hair of 13 Australian Brahman heifers. Since the Brahman breed shares ancestry with Nellore cattle, the ONT Brahman sequence set not only served as a validation set but also helped improve the resolution of structural variants via the use of long-read data. The ligation sequencing library preparation kit (SQK-LSK109) was used with a single R9.4.1 flow cell per animal on a MinION sequencer. Each flow cell was run for 96 h with two to three nuclease flushes to increase the flow cell yield. An average coverage of ~ 8.73$\times$ was obtained across all samples with a maximum of 13.6$\times$ and minimum of 6.21$\times$, and the average read length was 5.7 kb. Bases were called from fast5 signals using the Guppy software (v4.0.11, released August 2020, Oxford Nanopore Technologies). Long reads were aligned against the ARS-UCD1.2 bovine assembly using the Minimap2 software v2.14 [29] with the default ONT sequencing settings and the alignments were visualized with IGV [28].

### Annotation of transcripts

We used cDNA and annotation data from previous reports [30–33] to improve the annotation of functional candidate genes in the ARS-UCD1.2 bovine assembly. When coordinates for transcript elements (e.g., exons, introns, and UTR) were not available, cDNA sequences were aligned to the ARS-UCD1.2 bovine genome assembly to obtain approximate coordinates using BLASTN in Ensembl (available at: https://www.ensembl.org/Bos_taurus/Tools/Blast) and BLAST in NCBI (https://blast.ncbi.nlm.nih.gov/Blast.cgi).

### Estimation of coalescence time

We used the Genealogical Estimation of Variant Age (GEVA) method [34] to estimate the time of coalescence (in generation units) between carrier and non-carrier haplotypes of putative causal variants. This analysis was based on the 21 Nellore whole-genome sequences. Prior to the analysis, targeted chromosomes were filtered for bi-allelic variants presenting a minimum quality score of 60, a genotype quality score of 20, a call rate of 95% and a minimum allele count of 3, and then phased with the Eagle v2.4.1 software [22]. The effective population size, a parameter required by the GEVA method, was obtained from previously reported chromosome-specific estimates [35].

## Results

### GWAS maps DHC to chromosome 13 in Nellore cattle

**Fig. 1:**
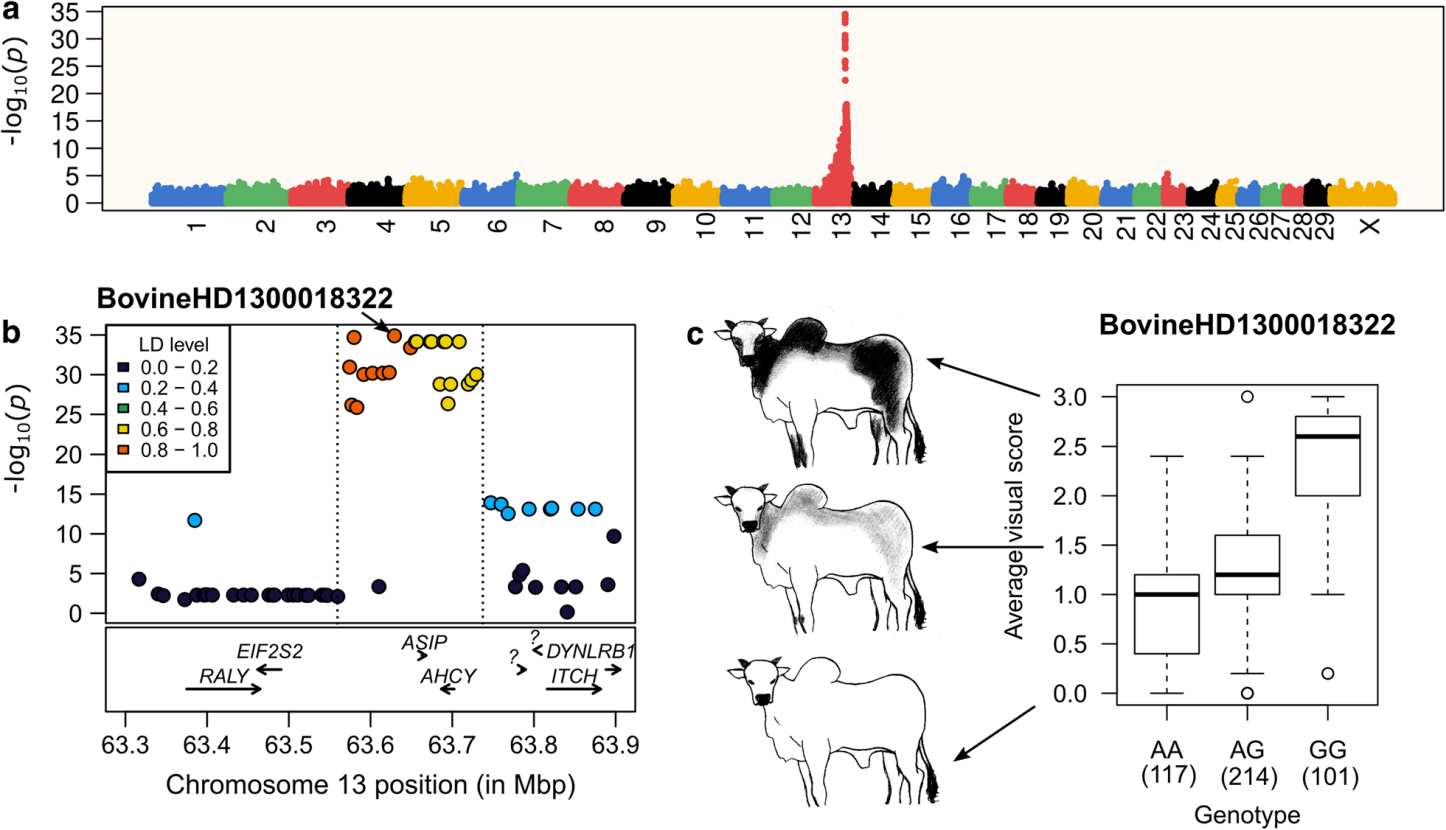
Genome-wide association analysis for DHC in Nellore cattle. **a** A single major locus mapping to chromosome 13 was detected (highest associated SNP, BovineHD1300018322; $p$ = 1.27 $\times$ 10^–35^). **b** Peak associations were found in a region (dashed lines) spanning the *ASIP* (CHR13:63,662,796–63,668,123) and *AHCY* (CHR13:63,686,723–63,702,437) genes. **c** The alternative G allele was correlated with increased darkness of hair coat in an additive pattern ($p$ = 1.42 $\times$ 10^–52^). Heterozygotes had their median phenotypic value skewed towards the median of the AA genotypic class, revealing a potential dominance effect associated with the reference A allele ($p$ = 1.84 $\times$ 10^–15^)

After filtering, 541,919 SNPs were screened for associations with DHC. The inflation factor was equal to 1.055, which indicates that the GWAS was properly corrected for relatedness and population substructure. A single GWAS hit was found on chromosome 13 (Fig. 1a). The most significant SNP, namely g.13:63,629,244A>G (rs109334889 or BovineHD1300018322, *p* = 1.27 × 10^–35^), had an alternative allele frequency of 48.1% in our sample, and was located approximately 33.5 kb upstream of the *ASIP* gene (Fig. 1b). This GWAS hit disappeared when we repeated the analysis by including this SNP as a fixed effect, which indicated that the signal was most likely driven by a single underlying causal variant or haplotype (see Additional file 2: Figure S2). Testing the marker for the presence of a dominance effect revealed estimates of $\alpha$ = 0702 ± 0.040 ($p$ =1.42 $\times$ 10^–52^) and $d$ = -0.467 ± 0.057 ($p$ = 1.84 $\times$ 10^–15^). This result suggests that DHC is associated with the alternative *G* allele in an additive pattern in Nellore cattle, but also that the reference *A* allele is related to a dominance deviation towards lighter coats than what would be expected for heterozygous animals on average (Fig. 1c). Furthermore, the leading SNP alone explained 46.6% of the variance in DHC.

**Fig. 2:**
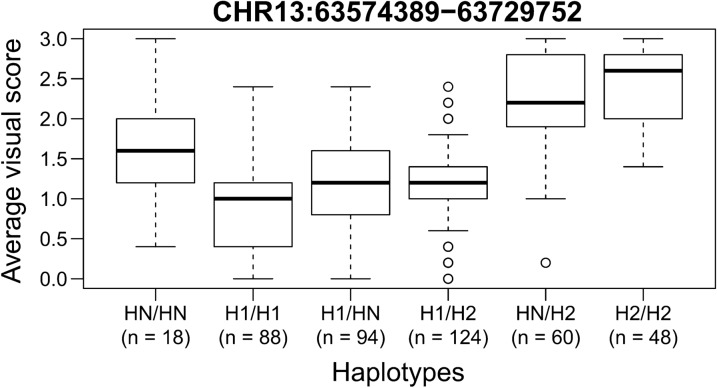
Phenotypic distribution conditional on haplotypes at the DHC association signal. Haplotype alleles were called on a block of 24 SNPs mapping to the association region on chromosome 13. Only two alleles had a frequency of at least 5%, namely H1 = TTGTATGTAACAATTGAAGGCCAA (frequency of 45.6%) and H2 = GCACGCGCGGTGGCCAGGAAATGG (frequency of 32.4%). The remaining 16 alleles were grouped in a single cluster (HN) for clarity. Alleles H1 and H2 exhibited contrasting phenotypic distributions, with H2 being involved with darker hair coat

We analyzed the haplotypic diversity in the chromosome 13 region that presented peak associations (CHR13:63,574,389–63,729,752) and included 24 SNPs (delimited by dashed lines in Fig. 1b). Eighteen haplotype alleles were identified, among which only two had a frequency higher than 5%. As expected, these two haplotypes, referred to as H1 (frequency of 45.6%) and H2 (frequency of 32.4%), had contrasting phenotypic distributions, with the H2 carriers tending to have a darker hair coat (Fig. 2). These results also support the hypothesis of a single underlying causal variant or haplotype driving the association signal at the candidate locus.

### Analysis of WGS data of Nellore bulls indicate that the causative variant is likely to have a regulatory effect

**Fig. 3:**
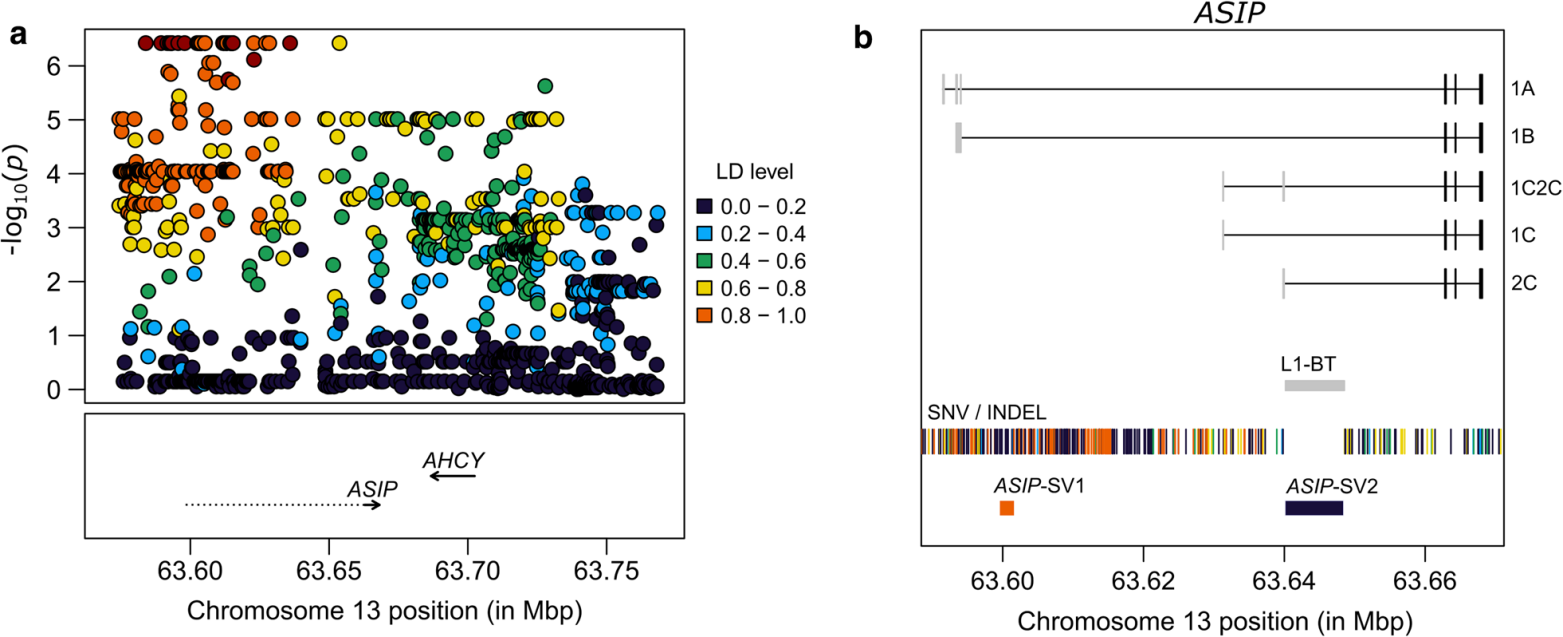
Regional association plots for DHC in 21 whole genome-sequenced Nellore bulls. **a** Peak associations were clustered upstream of the *ASIP* gene. The dashed line that extends upstream of the *ASIP* gene spans non-coding exons and introns that are not currently annotated in the ARS-UCD1.2 bovine genome assembly (Ensembl release 102). Linkage disequilibrium (LD) levels were calculated against the most significant variant in the region. **b** Details of the *ASIP* transcripts that differ in non-coding exons [33–35]. Single nucleotide variants (SNV) and small insertion/deletions (INDEL) are displayed as vertical bars, whereas structural variants (SV) are shown as rectangles. The long interspersed nuclear element (LINE) marked as L1-BT (*ASIP*-SV2) is responsible for a non-coding exon that is recruited by transcripts 2C and 1C2C. The first rectangle in the SV track (*ASIP*-SV1) is a 1155 bp deletion significantly associated with dark hair in the 21 sequenced animals ($p$ = 9.12 $\times$ 10^–5^)

In total, 1098 sequence variants that mapped to the region with a peak association (delimited by dashed lines in Fig. 1b) were extracted from WGS data of Nellore bulls. These included 932 intergenic, 65 intronic, 60 downstream, 32 upstream, five 3′-UTR, three synonymous, and one in-frame deletion variants in the *ASIP* gene (see Additional file 3: Table S1). In addition, two structural variants (SV) were found within the same chromosomal window. Using the phenotypes from the 21 sequenced bulls, we found that the variants located upstream of the *ASIP* gene were more strongly associated with DHC (Fig. 3a) than the other variants, which indicates that the underlying causal variant is less likely to be located in the coding regions of the gene. However, we found that an in-frame deletion that deleted the CGGACC sequence from the last exon of *ASIP* (rs519457228, located at 13:63,667,797–63,667,802) was a promising functional candidate. Unfortunately, it was poorly genotyped in our study, with a call rate of ~ 67%, suggesting alignment or sequencing issues. Further inspection of read alignments with IGV revealed that the coverage of the 17 WGS obtained from a previous study [14] was poor for that exon, which is GC-rich. This low coverage was most likely caused by PCR bias during library preparation, since these sequences were generated using legacy library preparation kits that are prone to amplification bias [36]. The sequence coverage drop was not observed in the four WGS generated with the TruSeq Nano kit for the current study, which supports our hypothesis of PCR bias. Thus, although rs519457228 was not included in the region with the strongest associations for DHC, we did not discard it as a candidate causal variant.

### Improved annotation of *ASIP* reveals alternative transcripts that recruit non-coding exons

Because we found strong associations that mapped upstream of the *ASIP* gene, we explored the presence of putative regulatory elements and additional exons and introns that could be missing in the current gene annotation. By manually curating previously described bovine *ASIP* transcripts [30–33], we found that the 5′-end of the *ASIP* gene is at least 71 kb longer than in the current annotation of the ARS-UCD1.2 bovine genome assembly (Ensembl release 102). Analysis of this additional sequence revealed that the top scoring upstream variants overlapped two bovine transcripts that recruit non-coding exons of *ASIP*, historically termed 1A and 1B (Fig. 3b). Due to limitations in predicting the functional impact of the candidate variants on these transcripts, we could not prioritize any particular SNV or small INDEL for further analysis, but they were retained as plausible positional candidates (see Additional file 3: Table S1). However, we did attempt to improve the resolution of the larger structural variants spanning the region, since they are more likely to impact gene expression and transcript diversity.

### A 1155-bp deletion that overlaps with alternative *ASIP* transcripts is associated with dark hair

One of the detected structural variants (hereafter referred to as *ASIP*-SV1) overlapped with the transcripts 1A and 1B and comprised a 1155-bp deletion spanning the region CHR13:63,599,803–63,600,957. The deletion allele had an estimated effect of 0.900 ± 0.182 ($p$ = 9.12 $\times$ 10^–5^) in the regression analysis, and thus correlated with darker hair. The second structural variant (hereafter referred to as *ASIP*-SV2) was also a deletion with respect to the reference genome, which spanned the L1-BT repeat (CHR13:63,639,817–63,648,206) known to recruit an additional non-coding exon in bovine transcripts 2C and 1C2C [31, 32]. However, the 21 Nellore bulls analyzed here lacked the L1-BT insertion. Therefore, *ASIP*-SV2 was less likely to affect DHC in Nellore cattle and consequently not further analyzed.

### The 1155-bp deletion serves as an insertion site for a duplicated inversion

**Fig. 4:**
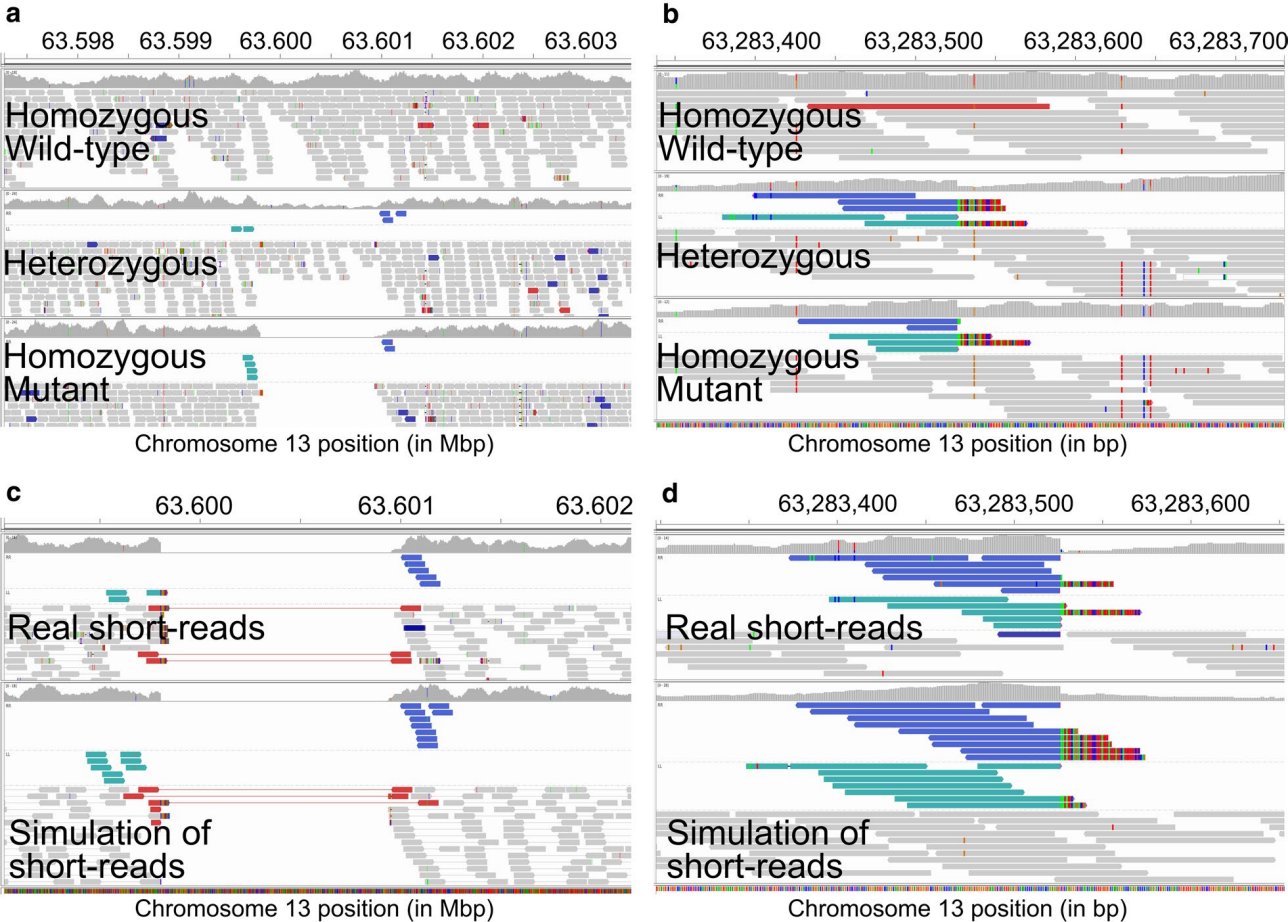
IGV screenshots for Illumina short-read alignments spanning a structural variant (*ASIP*-SV1) associated with DHC in Nellore cattle. **a** Presents an overview of the 1155 bp deletion at CHR13:63,599,803–63,600,957. Carriers presented read pairs with RR (cyan) and LL (blue) orientation flanking the deletion. **b** Shows a close inspection of RR and LL paired-end reads at the chromosome 13 position 13:63,283,374, revealing chimeric and soft- and hard-clipped reads. Simulations presented in **c** and **d** show that these alignments are consistent with the ~ 1-kb deletion being an anchoring point for the insertion of a reverse complement of the CHR13:63,283,374–63,283,523 sequence, which shares high similarity with the Bov-tA SINE. The wild type and mutant alleles were determined based on comparative genomics analyses presented in Additional file 5

Close inspection of *ASIP*-SV1 with IGV revealed the presence of read pairs that had an unexpected orientation (Fig. 4a). Whereas regular reads should be oriented inwards with respect to their inserts (RL orientation), for some bulls the read pairs had a single orientation, either pointing to the 3′ (RR orientation) or 5′ (LL orientation) end of the chromosome. In addition, the inserts for these single-orientation reads were typically longer than 316 kb, with paired-end reads mapping towards position CHR13:63,283,374 (Fig. 4b). Chimeric reads, as well as soft- and hard-clipped reads, were observed near the position CHR13:63,283,374. A BLAST analysis of the chimeric reads against the reference genome further confirmed that the foreign sequences belonged to the *ASIP*-SV1 region.

**Fig. 5:**
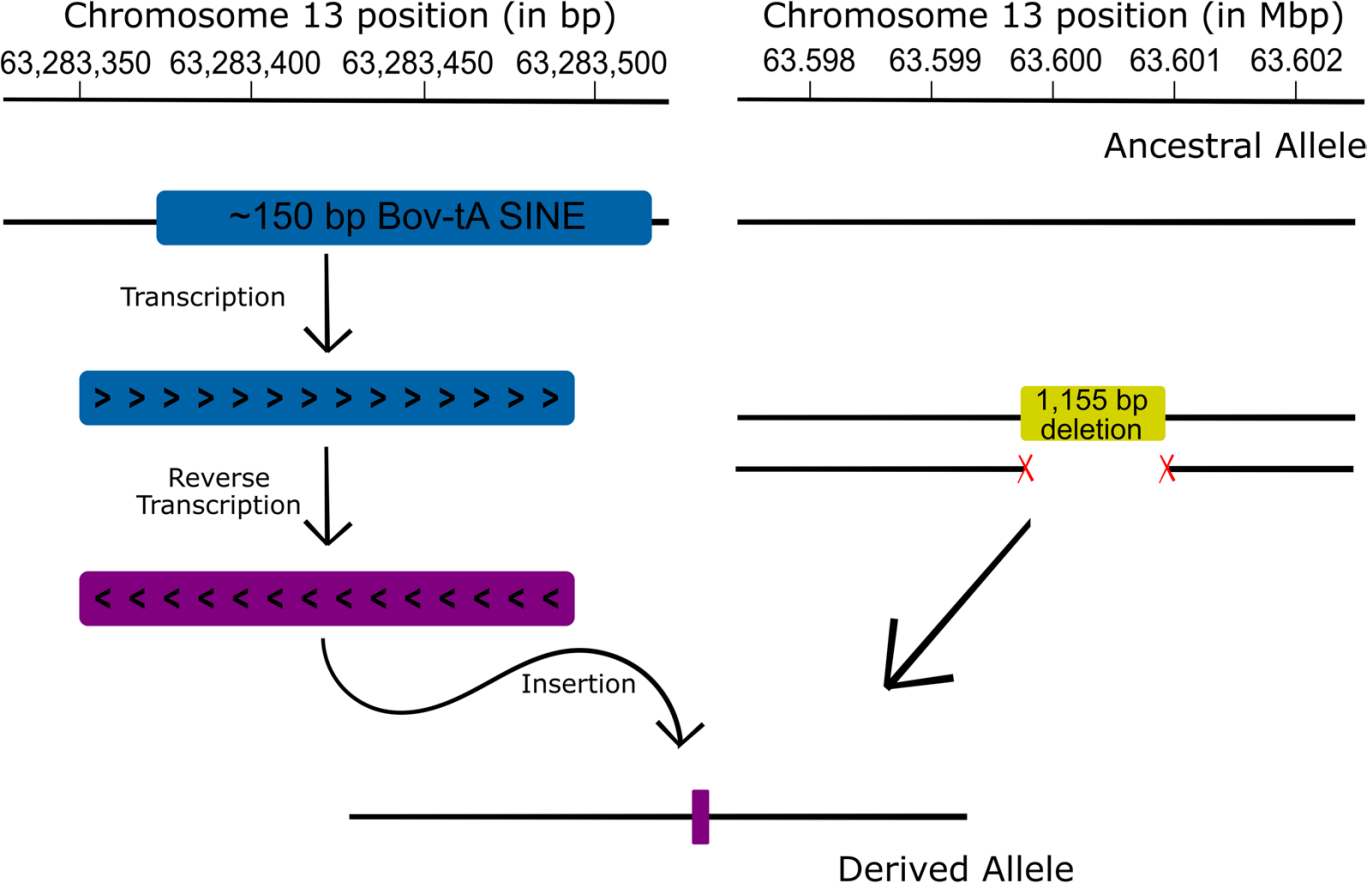
Schematic representation of the hypothesized mutation event leading to the structural variant (*ASIP*-SV1) associated with DHC in Nellore cattle. An expressed Bov-tA SINE was likely retrotranscribed and inserted in replacement of the 1155 bp sequence at CHR13:63,599,803–63,600,957

Taken together, our results suggest that the *ASIP*-SV1 region serves as an insertion site for an inverted duplication coming from CHR13:63,283,374. We performed several trial-and-error simulations of paired-end reads to find a structural rearrangement that could produce a good fit to the observed data. The best fitting simulation was a duplication of the CHR13:63,283,374–63,283,523 region, which had its reverse complement inserted at position CHR13:63,599,803 (Fig. 4c, d). We found that the inserted reverse complement had a match of 105 nucleotides with the Bov-tA SINE, which suggests that the inserted sequence was likely acquired through a retrotransposition event of an interspersed repeat (Fig. 5).

Using coalescent analysis, we estimated that the carrier and non-carrier haplotypes of this structural variant derived from a common ancestor that lived around 864 generations back, which translates into 3420 years ago assuming a generation interval of five years in cattle. Taking this estimate as a lower boundary for the age of the mutation, *ASIP*-SV1 is most likely an ancient derived allele that segregated in the *B. indicus* lineage. Therefore, *ASIP*-SV1 is expected to be present in other *B. indicus* breeds or even *B. taurus* populations that were historically introgressed with *B. indicus*.

### Analysis of nanopore sequence data of Brahman heifers refines the *ASIP*-SV1 structural variant

**Fig. 6:**
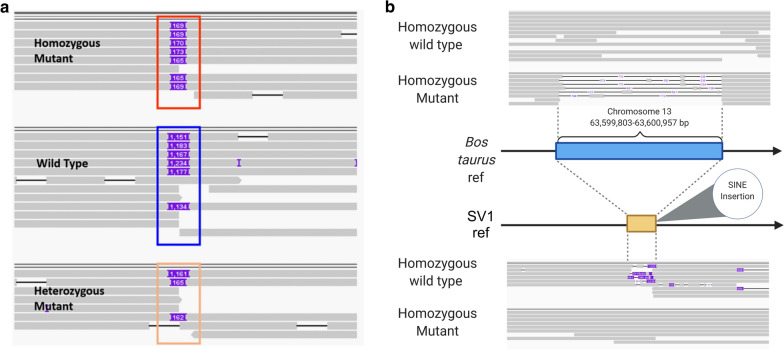
Oxford Nanopore Technologies long read alignments spanning *ASIP*-SV1 in Brahman cattle. **a** To improve visualization, the sequences were first aligned to a modified ARS-UCD1.2 assembly where the CHR13:63,599,803–63,600,957 segment was deleted. Wild type sequences are displayed as more than 1.1-kb insertions, whereas mutant sequences are displayed as insertions ranging from 162 to 173 bp (variation likely due to ONT sequencing errors). The only ONT read that mapped to the deletion position in the wild type animal was verified to be an alignment artifact (data not shown). **b** The long reads were further mapped to a modified ARS-UCD1.2 sequence that contained both the deletion and insertion of the reverse complement of CHR13:63,283,374–63,283,523

To confirm the *ASIP*-SV1 structural variant, we inspected the candidate region in ~ 8 × ONT data of 13 Brahman heifers from Australia (Fig. 6). Alignment of these long reads against the bovine genome assembly ARS-UCD1.2 revealed at least four carriers of the mutant haplotype. Due to the low genome coverage, we were unable to confidently determine the homozygous wild type, heterozygous and homozygous mutant genotypes across all the samples, but the list of the most likely genotypes are in Additional file 4: Table S2. Nevertheless, these data allowed us to confirm the existence of the *ASIP*-SV1 structural variant in Australian Brahman, and to verify that it segregates independently from rs519457228 (${r}^{2}$ = 0.04). By aligning the ONT reads against an augmented mutant genome in which the 1155-bp deletion was incorporated but not the insertion, we were able to infer that the inserted sequence is unlikely to be longer than 180 bp, although its exact sequence length remains unknown because of the high error rate related to single-molecule sequencing technologies.

## Discussion

In this paper, we report the identification of positional candidate variants that affect color patterns in Nellore (*Bos indicus*) cattle. Our study revealed a single major signal on chromosome 13 in the vicinity of the *ASIP* gene that is associated with DHC particularly on the head, neck, hump, and knees of male animals. Refinement of the signal with whole-genome short-read sequence data showed that the causal variant is likely to have a regulatory effect and is located in the 5′-region of *ASIP*, rather than affecting coding regions. This fits with the expectation that regulatory effects rather than changes to the protein are the cause of the observed quantitative pattern since alterations in the protein sequence encoded by autosomal genes would likely affect the whole coat qualitatively and would less likely behave in a sex-specific manner.

Among the possible causal variants, a complex structural rearrangement (*ASIP*-SV1) consisting of a 1155-bp deletion combined with an insertion of more than 150 bp including a SINE element seemed to be the most plausible candidate due to its size and location. The ancestral sequence was associated with lighter hair-coat, whereas the derived sequence was strongly correlated with darker hair. Furthermore, we showed that the *ASIP*-SV1 mutant haplotype segregates also in Australian Brahman cattle, and we were able to refine this *ASIP*-SV1 structural arrangement using ONT data generated for 13 Brahman heifers. In our study, we also found another important candidate variant, i.e. an in-frame deletion located in the last exon of the *ASIP* gene. Although this variant was not included in the segment with strong associations with DHC, it was poorly genotyped in our study due to PCR bias during library preparation and it could represent a sequencing or alignment artifact rather than a real variant. Since we were unable to discard it as a causal variant, it was retained as a functional candidate mutation. Studies in other species have already observed frameshift deletions that lead to uniformly eumelanistic (black) phenotypes [37–40]. However, a regulatory variant such as the *ASIP*-SV1 structural variant represents a more plausible candidate than a coding variant to explain the quantitative differences between light and dark Nellore bulls, as well as the specificity of the variation to certain parts of the body as opposed to the whole body.

The agouti signaling protein (ASIP) plays a crucial role in decreasing eumelanin and increasing pheomelanin production by blocking the melanocortin 1 receptor (MC1R) [41, 42]. ASIP controls the ratio of pheomelanin (yellow/red) to eumelanin (black) pigments and can act locally as an extracellular color modifier that influences the distribution of these pigments on the body [41, 42]. The primary ligand of MC1R is the α-melanocyte-stimulating hormone (α-MSH), which promotes eumelanin synthesis. ASIP acts as a competitive inhibitor of MC1R and promotes pheomelanin synthesis [40, 41], which means that decreased expression or loss of function of the *ASIP* gene would result in a black coat phenotype.

The bovine *ASIP* gene is composed of three coding exons (2, 3 and 4) and six additional 5′-UTR exons (1A, 2A, 3A, 1B, 1C and 2C) that, through permutation, can result in six transcripts (1A, 1B, 1C, 2C and 1C2C) that use different start sites [30–32]. The location of the *ASIP*-SV1 structural variant suggests that it may affect the expression of some, but not all, of these transcripts. An example of *ASIP* structural variants that impact the expression and diversity of the transcripts is found in Chen et al. [43], who report a ~ 3.1-kb element duplicated in reverse orientation that is located ~ 15 kb upstream of *ASIP* and causes a light-bellied phenotype in mice. Large structural variations involving the entire *ASIP* gene have also been shown to cause the white coat color in Merino sheep and Saanen goats [44–46]. Structural variations that affect only the 5′-regulatory region of *ASIP* are also responsible for three characteristic mutant coat patterns in goats [46], namely Swiss markings (*A*^*sm*^), badgerface (*A*^*b*^) and peacock (*A*^*pc*^), and for color dilution in quails [47].

Our findings indicate that the *ASIP*-SV1 structural variant was likely formed over 3000 years ago through a retrotransposition event of a Bov-tA SINE, which replaced a ~ 1-kb region of one of the cryptic introns of *ASIP*. Such retrotranspositions that affect host gene expression and transcript diversity, associated with variation in hair phenotypes, have been reported in other animal species. For example, Demars et al. [48] found that fleece variation in sheep was explained by the insertion of a *EIF2S2* antisense RNA into the 3′ UTR of *IRF2BP2*, leading to abnormal *IRF2BP2* transcription.

A recent study explored the genetics of white coat in buffaloes using whole-genome and RNA sequencing data [49]. The authors combined GWAS, biological experiments and population genomics to show that a ~ 2-kb LINE-1 insertion between exons 1C and 2 was responsible for an increased expression of *ASIP* and consequently for a white coat color. This insertion seems to act as a strong proximal promoter that increases the transcription of *ASIP* and affects melanocyte maturation. In contrast, our findings point to a ~ 1-kb deletion followed by a small SINE-1 insertion between the 1B and 1C non-coding exons of *ASIP* as the cause of dark hair on specific parts of the body in Nellore bulls. The two mutations are similar in the sense that they involve mobile DNA elements affecting alternative transcripts of *ASIP* that recruit non-coding exons. However, while the mutation reported here most likely increases eumelanin production, the mutation described in Liang et al. [49] seems to decrease it. In addition, the variant reported here shortens the regulatory sequence of the *ASIP* gene, whereas the variant in Liang et al. [49] lengthens it. These two mutations have reciprocal effects and provide further support that the candidate variant found here most likely affects one of the critical promoters of the *ASIP* gene.

A recent genetic divergence analysis conducted by Senczuk et al. [10] compared 15 white/grey Central and Eastern European *B. taurus* breeds with four non-grey northwestern cattle populations and identified three loci that displayed substantial divergence between the two groups, namely CHR2:6,510,630–7,010,630, CHR14:22,531,305–25,722,332 and CHR26:22,789,524–23,289,524. However, in our study, none of these loci were associated with DHC in Nellore cattle, which could be explained by one of the following alternative hypotheses: (a) the trait investigated differed between the studies, i.e. DHC on specific parts of the body analyzed in our work and white/grey hair as the predominant color of the animals’ body in the other study; (b) our study contained false negatives due to limitations in statistical power; (c) differences in the study design and data analysis could account for the different results, with one study performing GWAS on phenotypes measured in one breed and the other performing *F*_ST_ analysis without phenotypes in multiple breeds; (d) some of the loci reported by Senczuk et al. [10] are truly divergent between Central/Eastern and northwestern European breeds, but unrelated to coat color; or (e) different mutations affect the same phenotype in different breeds and sub-species.

Although we could not test the above-mentioned hypotheses directly, there is evidence in the Nellore data alone to support hypothesis (d) for the CHR14:22,531,305–25,722,332 region. Briefly, this chromosome 14 region has been shown to overlap with a large haplotype of northwestern *B. taurus* origin spanning the *pleomorphic adenoma gene 1* (*PLAG1*), which is associated with body size [14]. This haplotype is rare in Central and Eastern cattle, but segregates at high frequency (~ 18%) in Nellore cattle as a result of historical *B. taurus* introgression. Therefore, if the chromosome 14 region was strongly associated with white/grey hair, in our study we should have been able to identify an association signal for DHC on chromosome 14, or at least to detect non-grey hair-coat color in carrier bulls. Since neither of these possibilities was realized here, this chromosome 14 region is unlikely to contain variants that affect white/grey hair in the studied breeds.

Holland [12] analyzed the segregation of white/grey coat color in a Nellore-Angus crossbred population. Significant associations were found on chromosome 6 within an interval containing the *KIT* gene, which was previously implicated in coat color. As for the results by Senczuk et al. [10], we did not observe associations on chromosome 6 and this is most likely related to the above hypothesis (a), given that the traits under investigation differed between the two studies. Another possible explanation is that a variant in the *KIT* gene causes white/grey coat in Nellore cattle and the *ASIP* haplotype identified here further promotes dark hair on the head, neck, hump, and knees in males. Separate epigenetic mechanisms may underlie the red-to-black transition in pigmentation observed from birth to yearling and age-related darkening in males (e.g. methylation patterns, expression of miRNA and histone acetylation), and may also explain the sexual dimorphism in coat color (e.g. sex-specific *ASIP* regulatory elements, such as promoters and enhancers).

## Conclusions

We found a single statistically significant GWAS signal for darkness of hair coat in Nellore cattle, which mapped to the *ASIP* gene. A structural variant (*ASIP*-SV1) located upstream of *ASIP* was strongly associated with darker hair on the head, neck, hump and knee regions of males, which suggests that this variant is involved with decreased expression of *ASIP* and consequently a higher production of eumelanin. Although other candidate variants, including a 6-bp in-frame deletion in the last exon of *ASIP*, were not found in the region with the strongest association, they could not be excluded as causal variants. Overall, our study provides strong evidence that functional variants within or near the *ASIP* gene account for variation in regional darkness of hair coat in Nellore and Brahman cattle.

## Supplementary Information

**Additional file 1: Figure S1.** Histogram and summary statistics of average visual scores for darkness of hair coat in 432 Nellore bulls.

**Additional file 2: Figure S2.** Genome-wide association analysis for DHC in Nellore cattle including the BovineHD1300018322 SNP as a fixed effect.

**Additional file 3: Table S1.** List of positional candidate variants spanning the *ASIP* locus.

**Additional file 4: Table S2.** Inferred genotypes for *ASIP*-SV1 and rs519457228 in 13 Australian Brahman heifers sequenced with Oxford Nanopore Technologies.

**Additional file 5. **Additional methods—comparative genomics analysis to infer ancestral and derived *ASIP* alleles.

## Data Availability

The data used in this study were obtained under license and thus are not publicly available. However, the data sets are available for academic use from the corresponding author upon reasonable request.
