# Supplementary figures and images for: Variants at the ASIP locus contribute to coat color darkening in Nellore cattle
Source: Genet Sel Evol. 2021 Apr 28;53:40. doi: 10.1186/s12711-021-00633-2 (PMC8082809; doi:10.1186/s12711-021-00633-2)

## Darkness of hair coat

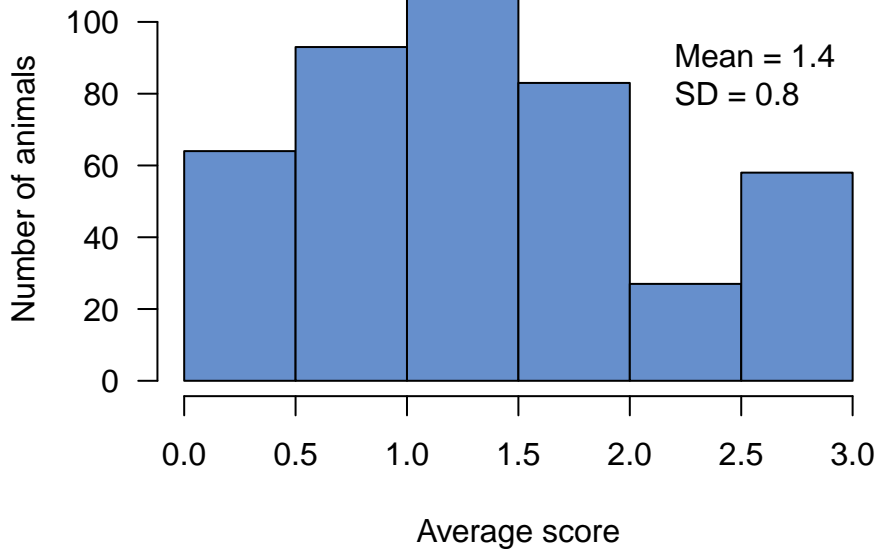

Supplement: Supplementary file 1 — Additional file 1: Figure S1. Histogram and summary statistics of average visual scores for darkness of hair coat in 432 Nellore bulls. [file 12711_2021_633_MOESM1_ESM.pdf]

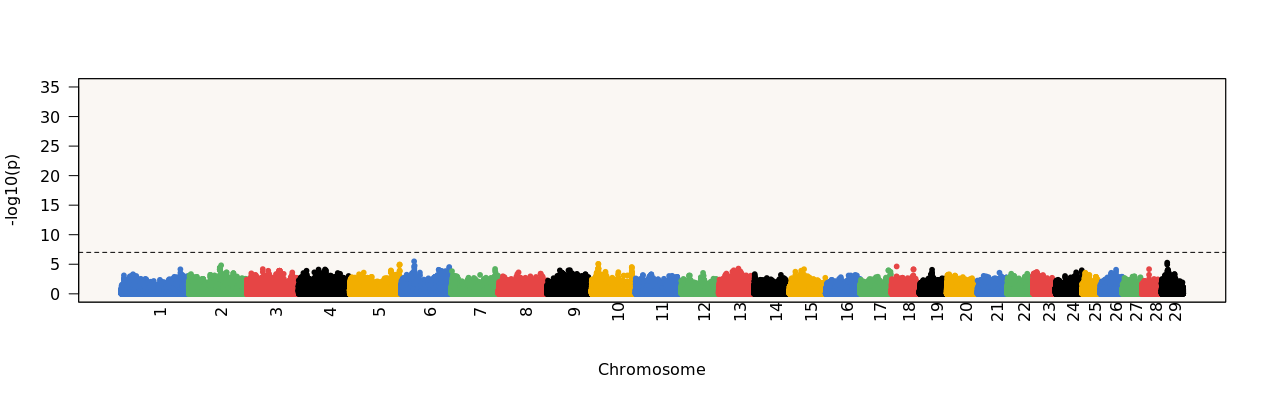

Supplement: Supplementary file 2 — Additional file 2: Figure S2. Genome-wide association analysis for DHC in Nellore cattle including the BovineHD1300018322 SNP as a fixed effect. [file 12711_2021_633_MOESM2_ESM.png]
