# Supplementary material for: Variants at the ASIP locus contribute to coat color darkening in Nellore cattle
Source: Genet Sel Evol. 2021 Apr 28;53:40. doi: 10.1186/s12711-021-00633-2 (PMC8082809; doi:10.1186/s12711-021-00633-2)
Supplement: Supplementary file 5 — Additional file 5. Additional methods—comparative genomics analysis to infer ancestral and derived ASIP alleles. [file 12711_2021_633_MOESM5_ESM.pdf]

ADDITIONAL METHODS

Variants at the *ASIP* locus contribute to coat color darkening in Nellore cattle

Beatriz Batista Trigo[1,2]; Adam Taiti Harth Utsunomiya[2,3]; Alvaro Augusto Anunziato Dock Fortunato[1,2,4]; Marco Milanesi[1,2,3]; Rafaela Beatriz Pintor Torrecilha[2,3]; Harrison Lamb[5]; Loan Nguyen[5]; Elizabeth M Ross[5]; Ben Hayes[5]; Rômulo Cláudio Morozini Padula[6]; Thayla Souza Sussai[6]; Ludmilla Balbo Zavarez[2]; Rafael Silva Cipriano[6]; Maria Margareth Theodoro Caminhas[1]; Flavia Lombardi Lopes[1]; Cassiano Pelle[7]; Tosso Leeb[8,9]; Danika Bannasch[8,10]; Derek Bickhart[11]; Timothy P L Smith[12]; Tad Stewart Sonstegard[13]; José Fernando Garcia[1,2,3,14]; Yuri Tani Utsunomiya[1,2,3]*

[1]São Paulo State University (Unesp). School of Veterinary Medicine, Araçatuba. Department of Production and Animal Health, São Paulo, Brazil; [2]International Atomic Energy Agency (IAEA) Collaborating Centre on Animal Genomics and Bioinformatics, Araçatuba, São Paulo, Brazil; [3]AgroPartners Consulting. R. Floriano Peixoto, 120 - Sala 43A - Centro, Araçatuba - SP, 16010-220; [4]Personal-PEC. R. Sebastião Lima, 1336 - Centro, Campo Grande - MS, 79004-600; [5]Centre for Animal Science, Queensland Alliance for Agriculture and Food Innovation, The University of Queensland, Australia; [6]Centro Universitário Católico Salesiano, Araçatuba, São Paulo, Brazil; [7]CRV Lagoa, Sertãozinho, São Paulo, Brazil; [8]Institute of Genetics, Vetsuisse-Faculty, University of Bern, Bremgartenstrasse 109A, 3012, Bern, Switzerland; [9]Dermfocus, University of Bern, Bremgartenstrasse 109A, 3012, Bern, Switzerland; [10]Department of Population Health and Reproduction, School of Veterinary Medicine, University of California, Davis, CA 95616, USA; [11]Dairy Forage Research Center, USDA-ARS, 1925 Linden Drive, Madison, WI, 53706, USA; [12]U.S. Meat Animal Research Center, USDA-ARS, 844 Road 313, Clay Center, NE 68933, USA; [13]Recombinetics Inc., St. Paul, MN, USA; [14]São Paulo State University (Unesp). School of Agriculture and Veterinarian Sciences, Jaboticabal. Department of Preventive Veterinary Medicine and Animal Reproduction, São Paulo, Brazil; *Corresponding author: ytutsunomiya@gmail.com

Additional methods

Comparative genomics analyses

In order to determine the ancestral and derived alleles at the *ASIP*-SV1 locus, we performed three separate analyses. In the first one, the bovine reference sequence (*Bos taurus* ARS-UCDv1.2) was aligned against the yak (*Bos grunniens* BosGru_v2.0), sheep (*Ovis aries* Oar_rambouillet_v1.0) and goat (*Capra hircus* ARS1) orthologous sequences using Clustal Omega v1.2.4 (available at https://www.ebi.ac.uk/Tools/msa/clustalo/). In the second one, FASTA files containing the whole bovine chromosome 13 sequence were aligned against the sheep and goat reference genome assemblies using blast2seq (available at https://blast.ncbi.nlm.nih.gov/Blast.cgi). One of the files had the bovine reference sequence unchanged, whereas the other one had the deletion at the *ASIP*-SV1 locus. Finally, simulated and real Oxford Nanopore Technologies (ONT) long reads containing the reference and alternative bovine *ASIP*-SV1 alleles were aligned against the yak genome using the alignment pipeline described in the article.

Results

The Clustal Omega alignments are displayed at the end of this file. They show that the bovine reference sequence at the *ASIP*-SV1 is also found in yak, sheep and goat (excluding small breakpoints of few bp), indicating that the 1,155 bp deletion associated with darker hair in Nellore cattle is a derived allele, whereas the reference bovine sequence is the ancestral state. **Figure SM1** shows the alignment of the whole bovine chromosome 13 – both in the reference and alternative forms – against the sheep and goat reference genomes, which further establishes the bovine reference as wild type and the deletion as mutant. Lastly, real and simulated ONT long reads were mapped against the yak genome (**Figure SM2**) to reinforce that the deletion associated with darker hair is the derived allele.

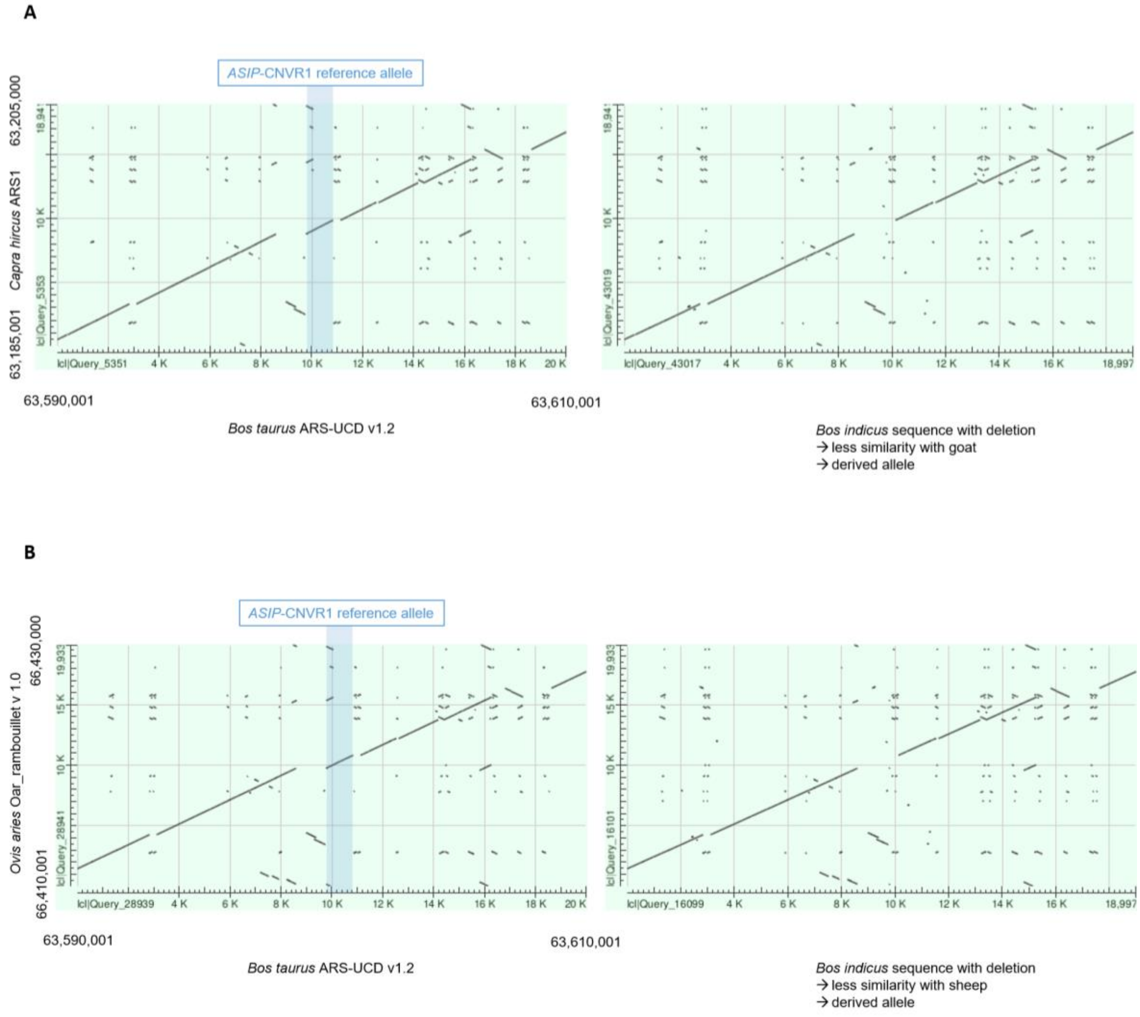

Figure SM1. Dot plots of bovine chromosome 13 alignments against the sheep (**A**) and goat (**B**) reference genomes. Both the reference (left side plots) and alternative (right side plots) bovine alleles were used. The reference allele is marked in blue. The plots show that the reference bovine allele is also found in sheep and goat, indicating that the deletion is the mutant sequence, and the reference allele is the wild type sequence.

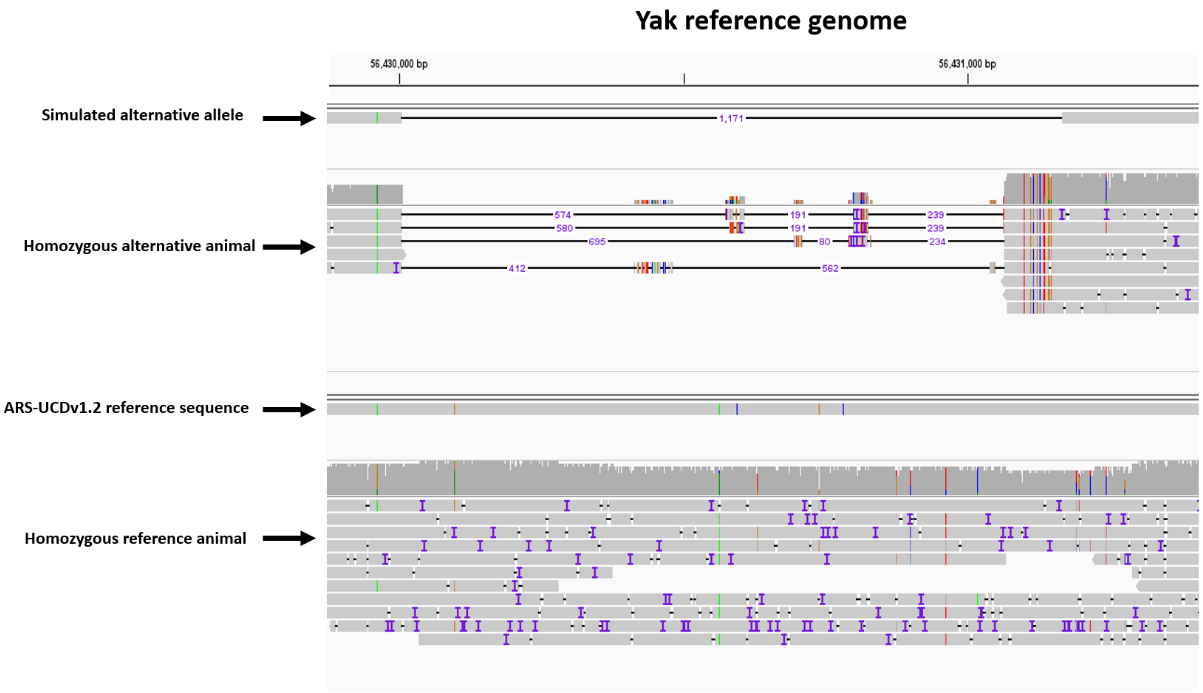

Figure SM2. Oxford Nanopore Technologies long reads mapped against the yak reference genome. Reads containing the bovine reference allele map through the yak sequence, whereas reads carrying the alternative allele display a ~1 kbp deletion at the *ASIP*-SV1 location. This indicates that the deletion is a derived sequence, whereas the bovine reference allele is ancestral.

```
CLUSTAL O(1.2.4) multiple sequence alignment

#OBS = The last ~200 bp were clipped due to breakpoints in sheep and goat

bos_taurus/11353                  ----TTGGAAGGACTGATGCTGAAGCTTCAATACTTTGGCCACCTGATGCAAACAGCTGA  56
bos_grunniens/11353               ----TTGGAAAGACTGATGCTGAAGCTTCAATACTTTGGCCACCTGATGCAAACAGCTGA  56
capra_hircus/11353                GGTAGGACTGATGCTGAAGCTGAAGCTTCAATACTTTGGCTACCTGATGCAAACAGCTGA  60
ovis_aries_rambouillet/11353      GGTAGGACTGATGCTGAAGCTGAAGCTTCAATACTTTGGCTACCTGATGCAAACAGCTGA  60
                                               **** ********************** *******************

bos_taurus/11353                  CACATTGGAAAAGGCCCTGATGGTGGGAAAGACTGAAGGCAGGAGGAGAAGGGGATGACA  116
bos_grunniens/11353               CACATTGGAAAAGGCCCTGATGGTGGGAAAGACTGAAAGCAGGAGGAGAAGGGGATGACA  116
capra_hircus/11353                CACATTGGAAAAGACCCTAACGCTGGGAAAGATTGAAGGCAGGAGGAGCAGGGGATGACA  120
ovis_aries_rambouillet/11353      CACATTGGAAAAGACCCTAATGCTAGGAAAGATTGAAGGCAGGAGGAGCAGGGGATGACG  120
                                  ************* **** * * * ******* **** ********** ********** 

bos_taurus/11353                  GAGGATGAGATGGTTGGATAACATCACCATTTCAATGGACATGAGTTTGAGCAAACTCTG  176
bos_grunniens/11353               GAGGATGAGATGGTTGGATAACATCACCATTTCAATGGACATGAGTTTGAGCAAACTCTG  176
capra_hircus/11353                GAGGATGAGATGGTTGGATGGCAACACCATTTCAATGGACATGAGCTTGAGCAAACTCTG  180
ovis_aries_rambouillet/11353      GAGGATGAGATGGTTGGATGGCATCACCATTTCAATGGACATGAGCTTGAGCAAACTCTG  180
                                  *******************  ** ********************* **************

bos_taurus/11353                  GGAGACAGTGAAGGACAGGGAAGCCCGGTGTGTTGCAGACCATGGGGTCACAAAGAGTCG  236
bos_grunniens/11353               GGAGACAGTGAAGGACAGGGAAGCCCGGTGTGTTGCAGACCATGGGGTCACAAAGAGTTG  236
capra_hircus/11353                GGAGTCAGTGAAGGACAGGGAAACCCAGTGTGTTGCAGACTATGGGATCACAAAGAACTG  240
ovis_aries_rambouillet/11353      GGAGGCAGTGAAGGACAGGGAAGCCCAGTGTGTTGCAGACTATGGGATCACAAAGAATTG  240
                                  **** ***************** *** ************* ***** *********   *

bos_taurus/11353                  GACATGACTTAGCAACTAAACTCACACAGTGTCCAGAGGAACTTGTGAAAAAGAAGCTTT  296
bos_grunniens/11353               GACATGACTTAGCAACTAAACTCACACAGTGTCCAGAGGAACTTGTGAAAAAGAAGCTTT  296
capra_hircus/11353                GACATGACTTAGCAACTAAACTCACACAGTGTACAGAGAAACTTGTGAAAAAGAAGCTTT  300
ovis_aries_rambouillet/11353      GACATGACTTAGCAACTAAACTCACACAGTGTACAGAGAAACTTGTGAAAAAGAAGCTTT  300
                                  ******************************** ***** *********************

bos_taurus/11353                  AAGGACCTAGATTCTGAGTCAT-AACTTGTAGAACTGAAGTGG------------GACCA  343
bos_grunniens/11353               AAGGACCTAGATTCTGAGTCAT-AACTTGTAGAACTGAAGTGG------------GACCA  343
capra_hircus/11353                AAGGACCTAGATTGTGAAGTCCTAACTTGCAGAACTGAAGTGGGACCGGGGGAATCTTTA  360
ovis_aries_rambouillet/11353      GAGGACCTAGATTGTGAAGTCCTAACTTGCAGAACTGAAGTGGGACCGGGGGAATCTTTA  360
                                   ************ ***      ****** *************                *

bos_taurus/11353                  GTGTTTCTTAAGGTGATGCTAATAAAAAAGATGCTCAGGAGACCCTTGGAGAAACATTTA  403
bos_grunniens/11353               GTGTTTCTTAAGGTGATGCTAATAAAAAAGATGCTCAGGAGACCCTTGGAGAAACATTTA  403
capra_hircus/11353                CCGTTTCTTAAGGTGATGCTAATAAAGAAGATGCTCAGGAGACCCTTGGAGAAACATTTA  420
ovis_aries_rambouillet/11353      CCATTTCTTAAGGTGATGCTAATAAAGAAGATGCTCAGGAGACCCTTGGAGAAACATTTA  420
                                     *********************** *********************************
```

```
bos_taurus/11353                   GAGAAAGAAACTGCCTGCTCAGGGAAGACTGGACTGATATCTGCATGTATGCCTTCTCCA  463
bos_grunniens/11353                GAGAAAGAAACTGCCTGCTCAGGGAAGACTGGACTGATATCTGCATGTATGCCTTCTCCA  463
capra_hircus/11353                 GAGAAAGAAACTACCTGCTCAGGGAAGACTGGACTGGTATCTGCATGTATGCCTTCTCCA  480
ovis_aries_rambouillet/11353       GAGGAAGAAACTACCTGCTCAGGGAAGACTGGACTGGTATCTGCATGTATGCCTTCTCCA  480
                                   *** ******** *********************** ***********************

bos_taurus/11353                   GATCCACGTGACTTTACCACACCTGGCCTCAGGACAGCACGAGCATTCTATCCCATCAAA  523
bos_grunniens/11353                GATCCACGTGACTTTACCACACCTGGCCTCAGGACAGCACGAGCATTCTATCCCATCAAA  523
capra_hircus/11353                 GATCCACATGACTTCACCACATCTGGCCTCAGGACAGCATGAGCATTCTATCCCATCAAA  540
ovis_aries_rambouillet/11353       GATCCACATGACTTCACCACATCTGGCCTCAGGACAGCACGAGCATTCTATCCCGTCAAA  540
                                   ******* ****** ****** ***************** ************** *****

bos_taurus/11353                   ATTATTCTGCTCCACTGGCTGTGAAGGAGAAGGAGAGATGCATGTTCCAGGACATTGGCT  583
bos_grunniens/11353                ATTATTCTGCTCCACTGGCTGTGAAGGAGAAGGAGAGATGCCTGTTCCAGGACATTGGCT  583
capra_hircus/11353                 GTTATTCTGCTCCACTGGCTGTGAAGGAGAAGGAGATACGCATGTTCCAGGACATTGGCT  600
ovis_aries_rambouillet/11353       GTTATTCTGCTCCACTGGCTGTGAAG---AAGGAGATACGCATGTTCCAGGACATTGGCT  597
                                    *************************   ******* * ** ******************

bos_taurus/11353                   CAGACAGGCCTACTGAGTGACTCTAAAGACTTGTAGACACCAAAAGGGTTTTTAAGAATA  643
bos_grunniens/11353                CAGACAGGCCTATTGAGTGACTCTAAAGACTTGTAGACACCAAAAGGGTTTTTAAGAATA  643
capra_hircus/11353                 CAGACAGGCCTACTGAGTGACTCTAAAGACTTGCAGACATCAAAAGGGTTTTGAAGAATA  660
ovis_aries_rambouillet/11353       TAGACAGGCCTACTGAGTGACTCTAAAGACTTGCAGACATCAAAAGGGTTTTGAAGAATA  657
                                    *********** ******************** ***** ************ *******

bos_taurus/11353                   ATCATCATAATATAGTAATGAATCAAAAACAAGCAGACATGTGCTTAAATGAGCCG-GAG  702
bos_grunniens/11353                ATCATCATAATATAGTAATGAATCAAAAACAAGCAGACATGTGCTTAAATGAGCCTGGAG  703
capra_hircus/11353                 TTCATCATAACATAGTAATGAATCAAAAACAGGCAGACATATGCTTAAATGAGCCTGGAG  720
ovis_aries_rambouillet/11353       TTCATCATAACATAGTAATGAATCAAAAACAGGCAGACATATGCTTAAATGAGCCTGGAG  717
                                    ********* ******************** ******** **************  ***

bos_taurus/11353                   GGACTGGAGTTAAAAGAAGGAAGGGGCTGTTCAGATTCGGGGGTCTAGCAGAATGGACTT  762
bos_grunniens/11353                GGACTGGAGTTAAAAGAAGGAAGGGGCTGTTCAGATTCAGGGGTCTAGCAGAATGGACTT  763
capra_hircus/11353                 GGACTGGAGTTAAAAGAAGGAAGGGGCTGTTCAGATTCAGGGGTCTAGCAGAATGGACTT  780
ovis_aries_rambouillet/11353       GGACTGGAGTTAAAAGAAGGAAGGGGCTGTTCAGACTCAGGGGTCTAGCAGAATGGACTT  777
                                   *********************************** ** *********************

bos_taurus/11353                   GGTTGAGAAAATCCAGCAAGACAACTGAAGAGGACTCTTGGGATTACCCCACCAGAACTT  822
bos_grunniens/11353                GGTTGAGAAAATCCAGCAAGATAACTGAAGAGGACTCTTGGGATTACCCCACCAGAACTT  823
capra_hircus/11353                 GCTTGAGAAAATCCAGCAAGACAACTGAAGAGGACTCTTGGGATTACCCCACCAGAACTT  840
ovis_aries_rambouillet/11353       GCTTGAGAAAATCCAGCAAGACAACTGAAGAGGACTCTTGGGATTACCCCACCAGAACTT  837
                                   * ******************* **************************************


bos_taurus/11353                   CTGAGGCAAGTCTAATGAAATGACTTACAAGTGTTAACTGTAGAGTAGCCTCTAAGGCGC  882
bos_grunniens/11353                CTGAGGCAAGTCTAATGAAATGACTTACAAGTGTTAACTGTAGAGTAGCCTCTAAGGCGC  883
capra_hircus/11353                 CTGAGGCAAGTCTAATGAAATGACTTACAAGTGTTAACTGTAGAGTAGCCGCTAAGGCGC  900
ovis_aries_rambouillet/11353       CTGAGGCAAGTCTAATGAAATGACTTACAATTGTTAACTGTAGAGTAGCCGCTAAGGCGC  897
                                   ****************************** ******************* *********

bos_taurus/11353                   CCCCATTGGCCTAATTCTGGTTGACATTGCACAACCAGGGTGAGCTGACA    932
bos_grunniens/11353                CCCCATTGGCCTAATTCTGGTTGACATTGCACAACCAGGGTGAGCTGACA    933
capra_hircus/11353                 CCACATTGGCCTAATTCTGGTTGACATTGCACAACCAGGGTGAGCTGACA    950
ovis_aries_rambouillet/11353       CCACATTGGCCTAATTCTGGTTGACATTGCACAACCAGGGTGAGCTGACA    947
                                   ** ***********************************************
```
